# EFTUD2 on innate immunity

**DOI:** 10.18632/oncotarget.5863

**Published:** 2015-09-28

**Authors:** Chuanlong Zhu, Fei Xiao, Wenyu Lin

**Affiliations:** Department of Medicine, Liver Center and Gastrointestinal Division, Massachusetts General Hospital, Harvard Medical School, Boston, MA, USA

**Keywords:** EFTUD2, HCV, RIG-I/MDA5, innate immunity, spliceosome, Immunology and Microbiology Section, Immune response, Immunity

Host innate immunity is the first line of defense against invasion of pathogens and determines the outcome of the infection. The host cells recognize pathogens as nonself via pattern-recognition receptors including Toll-like receptors (TLRs) and the RIG-I like receptors (RLRs), follow to induce local antiviral defense by producing interferon and proinflammatory cytokines to combat viral infection. Early innate immune response also stimulate adaptive immune to exert specific antiviral activity [1]. Hepatitis C virus (HCV) is a single-stranded RNA virus belonging to Flaviviridae family. Intrinsic innate immune clears HCV in 20% of primary infection. However, over 80% of infection evolves to chronic infection, which leads to hepatitis, liver cirrhosis and cancer. HCV, like other persistent viral infections, has evolved multiple strategies to disarm the innate immune system. For example, HCV non-structural protein NS3/4A blocks RIG-I and TLR signaling by cleaving mitochondrial antiviral signaling protein (MAVS), an interferon inducer, and a TLR3 adaptor protein, Toll/IL-1 receptor domain-containing adaptor inducing IFN-β (TRIF) [2]. Interaction of HCV evasion and host innate immune response determines the outcome of HCV primary infection.

On this study, Zhu *et al*. further explored HCV interaction with host cells intrinsic innate immune. These group investigators have identified elongation factor Tu GTP binding domain-containing protein 2 (EFTUD2) as a new host factor countering HCV infection [3, 4]. Moreover, they found that the spliceosome factor squamous cell carcinoma antigen recognized by T cells 1 (SART1), similar to EFTUD2, is not IFN-inducible but is an IFN effector gene (IEG). SART1 exerts its anti-HCV action through direct transcriptional regulation for some ISGs and alternative splicing for others ISGs [5]. EFTUD2 encodes a GTPase responsible for mRNA maturation and mutations. EFTUD2 has been reported to regulate the innate immune response through the alternative mRNA splicing of MyD88, a critical signaling adaptor in multiple Toll-like receptors (TLR) signaling pathways [6]. Zhu *et al.* have observed that EFTUD2 restricts HCV infection in Huh7 cells, but has no impact on HCV in RIG-I deficient Huh7.5.1 cells, indicating its anti-HCV effect is mediated by RIG-I. Silencing or overexpressing EFTUD2 results in down- or upregulated expression of RIG-I and MDA5, suggest that EFTUD2 controlled the expression of viral sensors RIG-I and MDA5 [4]. Interestingly, silencing EFTUD2 only reduces mature mRNA of RIG-I and MDA5 but the pre-mRNA, suggest EFTUD2 splices on viral sensor pre-mRNA [4]. In addition, RIG-I, MDA5, and EFTUD2 are found to regulate innate immune regulators interferon transcription factor 3 (IRF3) and TANK-binding kinase 1 (TBK1), and subsequently to induce interferon stimulated genes (ISGs) to exert antiviral activity. The authors also observed the lower protein expression of EFTUD2 in chronic HCV-infected liver biopsy compared to non-HCV biopsy [4]. We speculate that HCV suppresses EFTUD2 to its persistence. EFTUD2 and its downstream molecules RIG-I and MDA5 are able to sense both RNA viruses and some partially double-stranded DNA viruses such as hepatitis B virus. HBV uses a RNA proviral/intermediate-pregenomic RNA for replication. HBV infection triggers host innate recognition and responses. Recently, RIG-I has been identified dually functions as a sensor in activating innate signaling to counteract viral polymerase in HBV infection [7].

We therefore propose a unique model in which HCV and other RNA virus infection initiates the TLRs pathway signaling, which subsequently stimulates MyD88, this, in turn activates the phosphorylation of NF-κB. Activated NF-κB is then translocated to the nucleus to increase the production of anti-virus genes including TNF-α, IL6 and ISGs. EFTUD2 is a spliceosome factor which regulates RIG-I and MDA5 through mRNA splicing. HCV blocks large amount of ISGs production through specific inhibition of EFTUD2, RIG-I, and MDA5, and subsequently TBK1, IRF3 pathway to establish its persistent (Figure 1). Further understanding the mechanisms by which HCV perturbs host innate immune through EFTUD2 and its downstream genes to support its life cycle may provide novel antiviral strategies against HCV and other viruses. As one of the major spliceosome factors, EFTUD2 will be an interesting target to study the splicing mechanism by which EFTUD2 regulate on MyD88, RIG-I and MDA5. We anticipate novel findings to characterize RIG-I, MDA5, and MyD88 variants in HCV, or HBV-infected cells and patients.

**Figure 1:**
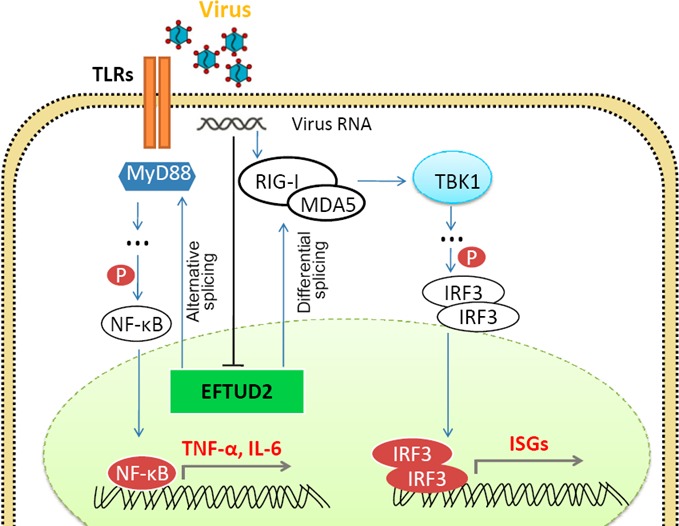
Proposed model of virus infection inhibits ISGs production through blocking EFTUD2 RIG-I, and MDA5 expression The initial virus infection activates host innate immune response to produce TNF-α, IL-6, and large amount of ISGs. EFTUD2 is one of a spliceosome factors which regulates RIG-I/MDA5 and MyD88 through mRNA splicing to activate the production of ISGs. Chronic HCV infection exerts its persistent through specifically reduces EFTUD2, RIG-I and MDA5 expression, and subsequently inhibiting TBK1, IRF3 phosphoylation and ISGs production.

## References

[1] Horner SM (2013). Nature medicine.

[2] Li K (2005). Proceedings of the National Academy of Sciences of the USA.

[3] Zhao H (2012). Journal of hepatology.

[4] Zhu C (2015). Journal of virology.

[5] Lin W (2015). Journal of hepatology.

[6] De Arras L (2014). Genetics.

[7] Sato S (2015). Immunity.

